# Study on the Strength Formation Mechanism of Coal Gangue-Based Composite Cementitious Materials

**DOI:** 10.3390/ma19101948

**Published:** 2026-05-09

**Authors:** Rongrong Feng, Xiaoxiao Wang, Feiquan Guo, Xiangdong Meng, Yuefei Lyu, Jie Liu

**Affiliations:** 1 College of Science, Inner Mongolia University of Technology, Hohhot 010051, China; 2 School of Civil Engineering, Inner Mongolia University of Technology, Hohhot 010051, China; 3 Inner Mongolia Key Laboratory of Green Construction and Intelligent Operation and Maintenance of Civil Engineering, Hohhot 010051, China; 4 Inner Mongolia Electric Power Construction (Group) Co., Ltd., Hohhot 010010, China; 5 China Huaneng Urad Middle Banner New Energy Power Generation Co., Ltd., Bayannur 015300, China

**Keywords:** coal gangue, water-to-binder ratio, pozzolanic reaction, compressive strength

## Abstract

Conventional cement production relies heavily on the consumption of natural resources, posing significant challenges to sustainable development. Incorporating activated coal gangue (CG) into cementitious materials not only reduces cement usage but also promotes the resource utilization of solid waste. In this study, the compressive strength (CS) and microstructural evolution of CG-based composite cementitious materials were systematically investigated under activated CG replacement levels of 0–50% and water-to-binder ratios (w/b) of 0.40–0.50. The results showed that the optimal performance was achieved at a CG replacement level of 30% and a w/b of 0.40, where the 28-day CS reached 59.1 MPa, representing an 18.9% improvement compared with the control group. The pozzolanic reaction of CG reduced the Ca(OH)_2_ (CH)content within the cementitious matrix and promoted the formation of ettringite (AFt) and C-(A)-S-H, thereby enhancing matrix compactness. These findings provide theoretical support for the application of CG in sustainable cementitious materials.

## 1. Introduction

Generated during coal mining and beneficiation, coal gangue (CG) makes up roughly 10–25% of total coal output [1,2]. CG is one of the most extensively generated industrial solid wastes, and its cumulative output from the coal industry has surpassed 7 billion tons, with annual growth reaching several hundred million tons [3,4]. Open-air stockpiling of CG not only consumes large areas of land, but also causes water contamination and dust emissions [5,6]. Heavy metal ions associated with CG can infiltrate and migrate with rainwater, thereby contaminating both groundwater and surface water. In addition, weathered and fragmented CG can generate fugitive dust, resulting in the deterioration of air quality in mining areas [7,8].

Current construction practices rely predominantly on Portland cement, the manufacture of which depends heavily on the extraction of natural resources. Using CG as a supplementary cementitious material in cement-based systems can reduce cement production costs, promote its value-added utilization, and lower the carbon footprint of cementitious materials. However, the principal mineral component of CG is kaolinite [9], which possesses a stable structure and relatively few defects; consequently, its chemical reactivity is low [10]. When directly incorporated into cement-based materials, CG generally acts more as an inert filler, resulting in low utilization efficiency and even deterioration in material performance [11,12]. Therefore, the key to the efficient utilization of CG lies in activating and regulating its mineral crystal structure and particle characteristics, thereby promoting the formation of amorphous phases and enhancing the reactivity of active silica and alumina so that it can exhibit higher pozzolanic activity. Currently, mechanical grinding [13] and thermal treatment [14] can render the clay minerals in CG more reactive, promoting the formation of pozzolanic phases such as SiO_2_ and Al_2_O_3_. For one thing, mechanical activation reduces the particle size of CG through grinding, thereby improving the packing density of the system via the micro-filling effect [15]. On the other hand, mechanical force induces lattice distortion, lowers mineral crystallinity, and increases the specific surface area, thereby generating more chemically active sites. Zhao et al. [16] mechanically activated CG by wet grinding and significantly enhanced its pozzolanic activity. As a supplementary cementitious material, activated CG accelerated cement setting and hardening, leading to an approximately 12% increase in compressive strength (CS).

Thermal activation, by contrast, promotes dehydroxylation and structural reorganization of clay minerals through calcination, thereby destroying the layered crystal structure and facilitating amorphization. In addition, it removes carbonaceous matter from CG, further enhancing its pozzolanic activity [14]. Guo Wei et al. [17] found that composite cement prepared from CG thermally activated at 700 °C, clinker, and gypsum exhibited relatively high mechanical performance. Activated CG promoted the early hydration of cement and facilitated the formation of C-S-H gel, Ca(OH)_2_ (CH), and ettringite (AFt). At a replacement level of 30%, the composite cement achieved CS of 20.3 MPa at 3 d and 40.1 MPa at 28 d. In addition, Cheng et al. [18] reported that, compared with Portland cement, the CG-cement composite cementitious system reduced carbon emissions by 7.9% and lowered cost by 5.3%. These findings indicate that replacing part of ordinary Portland cement with activated CG can not only effectively improve the strength of composite cementitious systems, but also reduce energy consumption.

The water-to-binder ratios (w/b) have a critical influence on the CS of cement-based materials [19,20]. According to Zhou et al. [21], the compressive strength of Ba(OH)_2_-activated hemihydrate phosphogypsum-based slag concrete varied with w/b in the order of 0.36 > 0.33 > 0.30 > 0.27 > 0.39. These findings suggest that, in general, a relatively low w/b is more favorable than a high one. However, a continuous reduction in the w/b may also be detrimental to CS [22,23]. This is because an excessively low w/b results in poor fluidity, leading to the formation of a large number of air voids during specimen preparation and molding, which weakens the matrix strength. Conversely, excessive water may also induce phase transformation during hydration due to excessive heat release, thereby promoting the formation of vapor pores [24]. Liu et al. [25] examined the mechanical behavior of alkali-activated composite cementitious materials with varying CG dosages and w/b, and found that the CS initially increased and then declined as the CG content increased. However, the optimal peak strength did not occur at the same point under different w/b. A possible reason is that excessive CG absorbs more mixing water, thereby limiting gel formation and ultimately lowering the overall CS. As the w/b increases, more water becomes available to CG, thereby mitigating its adverse effect on strength. Collectively, these observations suggest that it can be concluded that the mechanism by which the w/b affects CS varies among different cementitious systems.

While the influence of activated CG on the mechanical performance of composite cementitious materials has been widely studied, the mechanism by which activated CG dosage and w/b jointly affect strength development has not yet been fully clarified. Therefore, this study systematically examines how activated CG dosage and w/b influence the mechanical properties and hydration behavior of composite cementitious materials. Particular attention is given to the evolution of hydration products, the consumption of CH, and the development of microstructure, with the aim of clarifying the mechanism responsible for strength improvement in CG-cement composite systems.

## 2. Materials and Methods

### 2.1. Materials

**Figure 1:**
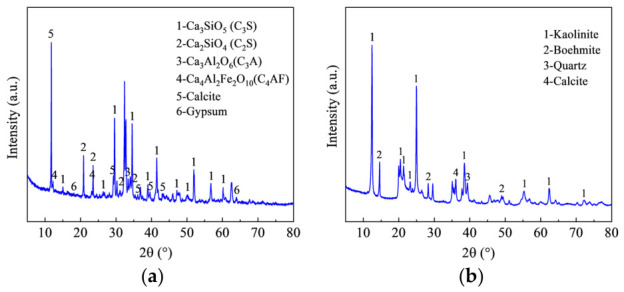
XRD patterns of CG and cement. (**a**) Cement. (**b**) CG.

P.O 42.5 ordinary Portland cement supplied by Inner Mongolia Mengxi Cement Co., Ltd. (Ordos City, China) was used in this work, and its chemical composition is listed in Table 1. Calcium oxide was the predominant chemical component, accounting for 46.72 wt.%. The principal mineral phases in the cement were gypsum (CaSO_4_·2H_2_O), tricalcium aluminate (Ca_3_Al_3_O_6_, C_3_A), dicalcium silicate (Ca_2_SiO_4_, C_2_S), tetracalcium aluminoferrite (Ca_4_Al_2_Fe_2_O_10_, C_4_AF), calcite (CaCO_3_), and tricalcium silicate (Ca_3_SiO_5_, C_3_S), as shown in Figure 1a.

The coal gangue (CG) was collected from a mining area in Ordos, Inner Mongolia, and its chemical composition is also listed in Table 1. The mineralogical composition of the CG is presented in Figure 1b. According to Table 1, the major chemical constituents of the CG were silica and alumina, with mass fractions of 43.63 wt.% and 48.04 wt.%, respectively. The main mineral phases of the CG were kaolinite (Al_4_[Si_4_O_10_]_8_), boehmite (AlOOH), quartz (SiO_2_), and calcite (CaCO_3_).

**Figure 2:**
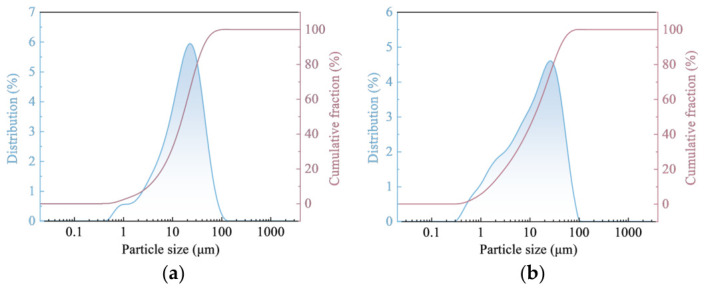
Particle size distribution of CG and cement. (**a**) Cement. (**b**) CG.

The particle size characteristics of cement and CG are shown in Figure 2. CG exhibited a main particle size range of 0.357~98 μm, while that of cement was 0.523~111 μm. Particle size parameters are provided in Table 2.

**Figure 3:**
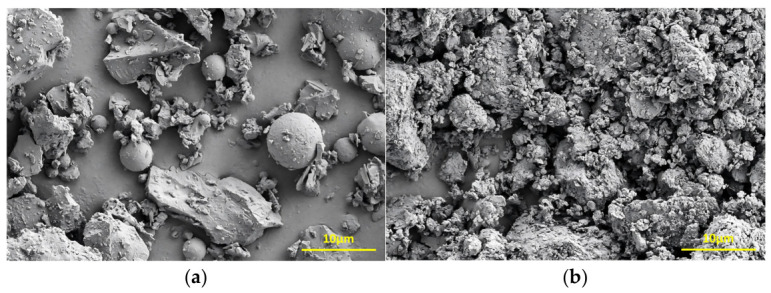
SEM image of cement and CG. (**a**) Cement. (**b**) CG.

The micromorphologies of the cement and CG samples were examined, as shown in Figure 3. The cement consisted of irregular angular particles and spherical particles with varying sizes (Figure 3a). In contrast, the CG sample contained irregular block-like particles, on the surfaces of which flocculent and disordered amorphous substances were attached (Figure 3b).

### 2.2. Activation Process of Coal Gangue

The CG was subjected to the two-stage activation treatment. The first stage was mechanical activation, and the procedure was as follows. First, the CG was crushed into particles with sizes of 5~10 mm using the TJEP-100 × 60 crusher (Hebi Tianjian Electronic Technology Co., Ltd., Hebi, China). The crushed particles were then ground in an SM500 × 500 ball mill for 2 h to obtain CG powder. The ground powder was subsequently sieved using an S49-AC-800 ultrasonic vibrating sieve machine (Xinxiang Gaofu Machinery Co., Ltd., Xinxiang, China) equipped with a square-hole sieve of 0.075 mm aperture. Finally, the sieved CG was dried in a DHG-9023A forced-air drying oven at 105 °C for 6 h to obtain mechanically activated CG.

**Figure 4:**
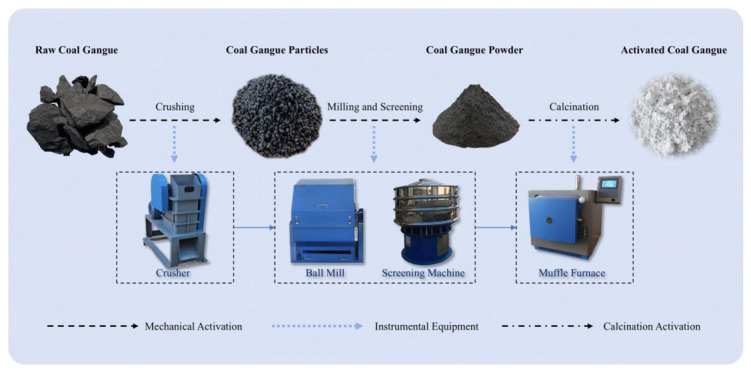
Flowchart of the activation treatment process for CG.

The second stage was thermal activation. Specifically, the dried CG was placed in a JZXL9000A (Hebi Jingke Technology Co., Ltd., Hebi, China) muffle furnace for calcination. The calcination program involved heating the sample from room temperature to the target calcination temperature at the rate of 10 °C/min, followed by holding for 2 h. After calcination, the CG was promptly taken out of the muffle furnace and cooled naturally to room temperature, resulting in the two-step activated CG powder. The overall preparation route is illustrated in Figure 4.

### 2.3. Mix Proportion

**Figure 5:**
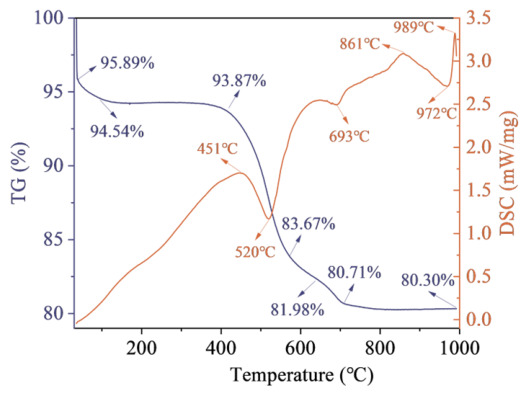
TG/DSC curve of CG.

Figure 5 presents the thermogravimetric–differential scanning calorimetry (TG/DSC) curves of the CG. As shown in the figure, the total mass loss of the sample from room temperature to 1000 °C is 15.59%. When the calcination temperature is within 20~400 °C, the mass loss is 2.02%, which is primarily attributed to the evaporation of a small amount of free water and the combustion of trace organic matter contained in the CG. In the temperature range of 400~700 °C, the mass loss reaches 13.16%, mainly due to the dehydroxylation of kaolinite (Al_2_Si_2_O_5_(OH)_4_). Previous studies [26] have shown that calcination leads to the breakdown of the kaolinite lattice and the formation of amorphous metakaolin (Al_2_Si_2_O_7_), a highly reactive pozzolanic phase. This transformation increases the contents of amorphous SiO_2_ and Al_2_O_3_, thereby enhancing the reactivity of the CG.

Based on the analysis of the thermogravimetric curve of coal gangue and the discussion of the optimal calcination temperature reported in references [27,28], this study employed coal gangue activated by calcination at 700 °C for 2 h to prepare the CG-based composite cementitious materials (CGCC). The effects of the w/b (0.40, 0.45, and 0.50) and the dosage of activated CG (0%, 10%, 20%, 30%, 40%, and 50%) on the mechanical properties, hydration characteristics, and microstructure of the CGCC were systematically investigated. On this basis, the strength enhancement mechanism of the CGCC was further elucidated. The detailed mix proportions are presented in Table 3, where the contents of CG and cement are expressed as mass fractions. Distilled water was used in specimen preparation to prevent extraneous ions in tap water, such as Ca^2+^, Mg^2+^, Na^+^, K^+^, Cl^−^, SO_4_^2−^, and HCO_3_^−^ from interfering with the hydration process of the CGCC.

### 2.4. Test Methods

#### 2.4.1. Setting Time Test

The setting time was determined in accordance with GB/T 1346-2024 [29]. The setting behavior of CGCC with CG replacement levels of 0%, 10%, 20%, 30%, 40%, and 50% and w/b of 0.40, 0.45, and 0.50 was evaluated using a Vicat apparatus (Wuxi Zhongke Building Materials Instrument Co., Ltd.: Wuxi, China).

#### 2.4.2. Compressive Strength Test

The compressive strength (CS) of the CGCCs was tested using a Sinter YAS-600 testing Machine (Changchun New Testing Machine Co., Ltd.: Changchun, China). A loading rate of 2.4 kN/s was applied. For each group, six specimens were tested, and the mean value was taken as the CS. The crushed CGCC obtained after the compressive strength test was collected and immersed in absolute ethanol to stop hydration. Before conducting the microstructural analyses, the samples were dried at 45 °C for 24 h, followed by grinding and sieving through a 0.075 mm mesh.

#### 2.4.3. XRD Test

XRD characterization was carried out on a DX-2700B diffractometer (Dandong Haoyuan Instrument Co., Ltd., Dandong, China), and the diffraction data were processed with HighScore (Plus) 5.3.0 software. X-ray diffraction analysis was performed using a DX-2700B diffractometer. Cu-Kα radiation was employed with an operating voltage of 40 kV and a tube current of 30 mA. The samples were scanned at a speed of 6°/min over a 2θ range of 5~90°, with a step size of 0.02°. For sample preparation, specimens cured to the designated ages were immersed in absolute ethanol to terminate hydration. Prior to testing, the samples were removed from ethanol, oven dried at 45 °C for 24 h, and then ground to a fine powder. The powder was subsequently sieved through a 0.075 mm mesh to ensure uniform particle size, and then pressed into a flat specimen holder to obtain a smooth surface for analysis. Phase identification was conducted using HighScore software after data acquisition.

#### 2.4.4. FTIR Test

FTIR analysis was performed using an IRTracer-100 Fourier-transform (Shimadzu Enterprise Management Co., Ltd., Beijing, China) infrared spectrometer. Prior to testing, the samples were ground into fine powder and passed through a 0.075 mm sieve. The sieved powders were then dried in a forced-air oven at 45 °C for 24 h. After drying, the powders were thoroughly mixed with spectroscopic-grade KBr at a mass ratio of 1:100 and further ground using an agate mortar. The mixture was subsequently pressed into transparent pellets using a tablet press. FTIR spectra were collected over the wavenumber range of 4000~400 cm^−1^. Peak identification and functional group analysis were conducted using OMNIC 8.2.0 software.

#### 2.4.5. TG/DTG Test

Thermogravimetric analysis of CGCC was conducted using a HITACHI STA 200 (Hitachi, Ltd., Tokyo, Japan) simultaneous thermal analyzer. After hydration was terminated, the samples were dried in an oven at 45 °C for 24 h, ground into powder, and passed through a 0.075 mm sieve. A sample mass of approximately 15 mg was accurately weighed using a precision electronic balance and placed into a small crucible for testing. Before measurement, the instrument was preheated for 24 h. The crucible was then placed into the analyzer, and the heating program was set with a heating rate of 10 °C/min, starting from 25 °C and increasing to 800 °C. Nitrogen was used as the protective atmosphere during the test. The instrument recorded the TG signal and subsequently generated the DTG curve. The obtained results were used to analyze the mass loss characteristics of the samples within different temperature ranges and to identify the corresponding thermal decomposition behaviors of various phases.

#### 2.4.6. SEM Test

Microstructural examination of the raw materials and heat-treated CG powders was carried out using an FEI Apreo 2S field-emission SEM (Thermo Fisher Scientific Inc., Waltham, MA, USA). The samples were attached to conductive adhesive, sputter-coated with gold, and observed at 3 kV.

#### 2.4.7. Hydration Heat Test

The hydration heat of CGCC with different mix proportions was measured using a TAM Air eight-channel isothermal calorimeter (TA Instruments, Inc., New Castle, DE, USA). The testing temperature was maintained at 25 °C. Prior to the test, the raw materials of the binder for each mixture were weighed using an analytical balance with an accuracy of 0.1 mg. The total mass of the powder sample and distilled water was fixed at 6 g. The weighed materials were placed into a glass ampoule and mixed using a mechanical shaker for 1 min to ensure uniformity. Immediately after mixing, the ampoule was transferred into the calorimeter channel, minimizing the time interval between sample preparation and the start of measurement so as to reduce the influence of early hydration heat loss on the results. After the instrument reached a stable operating state, data acquisition was initiated. The hydration behavior of CGCC with different mix proportions was recorded continuously for 168 h.

#### 2.4.8. Pore Structure Test

A MesoMR23-060H-I nuclear magnetic resonance (NMR) analyzer (Suzhou Niumai Analytical Instrument Co., Ltd., Suzhou, China) was employed to characterize the pore structure of CGCC samples with different mix proportions. Before measurement, the specimens were saturated in a vacuum saturation device for 24 h and then placed inside the testing coil. The NMR analyzer was subsequently used to determine parameters such as porosity and pore size distribution of the CGCC samples.

## 3. Results

### 3.1. Compressive Strength

**Figure 6:**
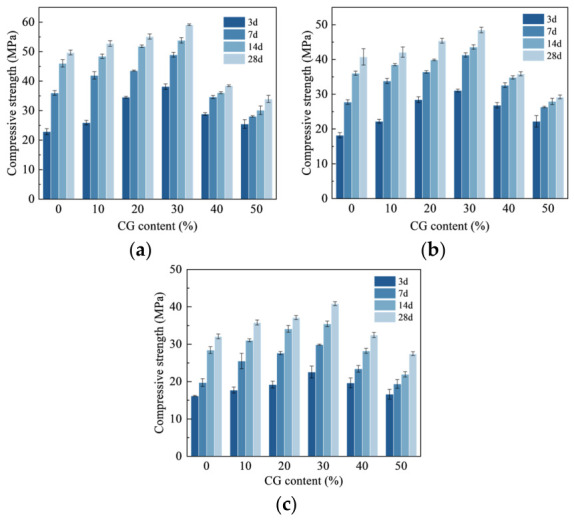
CS of CGCC at different w/b. (**a**) 0.4. (**b**) 0.45. (**c**) 0.5.

Figure 6 illustrates the variation in the CS of CGCC with the w/b. When the w/b was 0.50, the CS of the ordinary Portland cement paste at 28 d was 32.1 MPa. When the w/b decreased to 0.40, the CS increased to 49.7 MPa, corresponding to an increase of 54.8%. For the composite cementitious material containing 30% CG, compared with the specimen prepared at a w/b of 0.50, the 28 d CS increased to 48.5 and 59.1 MPa when w/b was reduced to 0.45 and 0.40, respectively, representing increases of 18.6% and 44.4%. One possible reason is that cement hydration requires a nearly fixed amount of water per unit volume [30]. A higher w/b introduces more free water into the system. After setting and hardening, the loss of this excess water produces more pores and weakens matrix compactness. Meanwhile, greater particle dispersion at high water content may retard cement hydration and hinder the rapid formation of a continuous strength-bearing skeleton [31].

At a w/b of 0.40, the maximum 28 d CS of 59.1 MPa was achieved at 30% CG. Compared with the reference group without CG, the 28 d CS increased by 6.2%, 10.9%, and 18.9% at CG contents of 10%, 20%, and 30%, respectively. The strength gain was closely related to the alkaline conditions established by Ca(OH)_2_ (CH) from cement hydration, under which the reactive SiO_2_ and Al_2_O_3_ in CG became involved in pozzolanic reactions. These hydration products contributed to pore filling and matrix densification. Nevertheless, further increasing the CG content to 40% and 50% caused a pronounced decline in CS, with the 28 d values falling to 38.5 MPa and 34.0 MPa, respectively, i.e., 53.5% and 73.8% lower than that of the 30% CG specimen. A possible explanation for this decline is that the reduced cement content limited the supply of CH. Under this condition, a larger fraction of CG remained unreacted, while the generation of binding phases became insufficient, ultimately hindering matrix strength formation.

### 3.2. Setting Time

**Figure 7:**
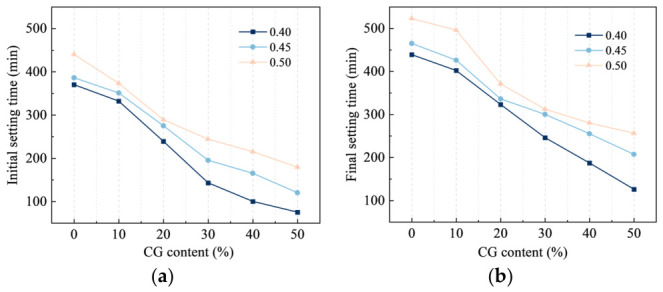
Setting times of CGCC. (**a**) Initial setting time. (**b**) Final setting time.

Figure 7 shows the variation in the setting time of CGCC at different w/b. A pronounced shortening of setting time occurred when the w/b was reduced from 0.50 to 0.40. In the 0% CG system, the initial and final setting times decreased from 440 to 370 min and from 523 to 439 min, respectively, whereas in the 30% CG sample, they declined from 244 to 143 min and from 312 to 246 min. At 50% CG, these values were further reduced to 75 min and 126 min, indicating substantial shortening of the setting process. The setting behavior of cementitious materials has been reported to be closely related to hydration rate and hydrate generation [32]. At a lower w/b, the spacing between cement particles and CG particles is reduced, which facilitates the formation of a structural skeleton and thereby shortens the setting time. A higher w/b leads to a more porous system, which increases the demand for hydration products to fill the matrix and thus slows down setting and hardening [33].

With increasing CG content, the setting time of the CGCC was significantly shortened. Specifically, the reductions in initial setting time were 38, 131, 227, 270, and 295 min at CG contents of 10%, 20%, 30%, 40%, and 50%, respectively, whereas the corresponding reductions in final setting time were 37, 116, 193, 252, and 313 min. Experimental results indicated that the activated CG possessed a larger specific surface area. As a result, CG particles could interact more readily with water during hydration, which increased the effective reaction interface and promoted the hydration process of the CGCC. In addition, the activation treatment increased the number of particle defects and intensified lattice distortion in the CG, resulting in reduced structural stability and enhanced potential reactivity. In the hydrating matrix, these changes promoted structural reorganization and hydration product formation, thereby speeding up the setting and hardening process [34]. Calcination also made the CG structure looser and more porous, which increased its water absorption capacity and reduced the fluidity of the cementitious system.

## 4. Discussion

### 4.1. Hydration Heat Analysis

**Figure 8:**
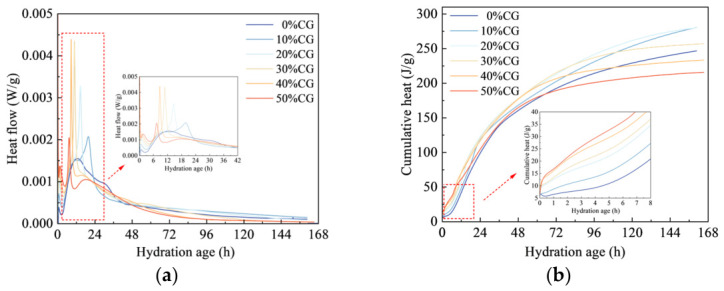
Hydration heat evolution behavior of CGCC with different CG contents. (**a**) Hydration heat evolution curve. (**b**) Cumulative hydration heat evolution curve.

The heat flow profiles of cement pastes with different CG contents over 168 h are shown in Figure 8a. When the hydration age exceeded 24 h, the heat evolution curves gradually became stable, indicating that the chemical reactions proceeded at a relatively slow rate at this stage. During the first 6 h of hydration, the cumulative heat output increased progressively with increasing activated CG dosage. In addition, the first exothermic peak gradually increased in magnitude and shifted progressively to a later time with increasing activated CG dosage. This suggests that the presence of CG retarded the early dissolution of cement particles and extended the induction stage of hydration. For cement pastes containing 0%, 10%, 20%, 30%, 40%, and 50% activated CG, the first exothermic peak occurred at 43, 43, 44, 55, 67, and 96 min, respectively, with corresponding heat flow values of 0.389, 0.717, 1.027, 1.211, 1.351, and 1.373 mW/g, respectively. These findings suggest that activated CG delayed the initial heat release of cement paste hydration. This is likely due to the reduction in cement content after CG incorporation, which lowered the contents of minerals such as C_3_A, C_4_AF, and gypsum in the system, thereby reducing the initial dissolution heat. Further observation of the second exothermic peak in the hydration period of 6–36 h showed that, with increasing CG content, the hydration heat evolution peak gradually shifted to an earlier time. This behavior suggests that activated CG at higher dosages accelerated overall hydration by offering more surfaces for hydrate nucleation and growth. During the deceleration stage, reactive SiO_2_ and Al_2_O_3_ in activated CG further consumed CH from cement hydration, promoting secondary reactions and speeding up overall hydration [35].

**Figure 9:**
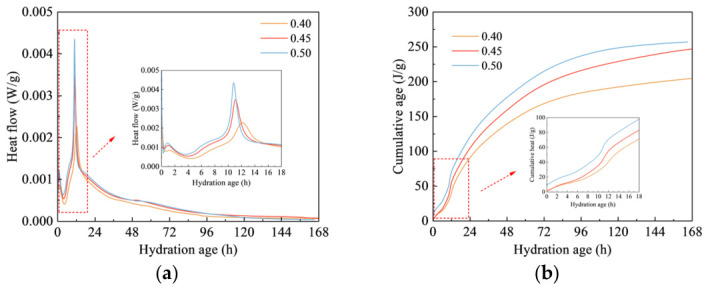
Heat evolution curves of the CGCC with different w/b. (**a**) Hydration heat evolution curve. (**b**) Cumulative hydration heat evolution curve.

Figure 9 shows the hydration heat evolution of the 30% CG sample under different w/b. With increasing w/b, the main heat-release peak shifted toward an earlier stage and increased in magnitude. When the w/b was 0.40, 0.45, and 0.50, the exothermic peak appeared at 12.3, 11.1, and 10.85 h, respectively, with corresponding peak heat flow values of 0.0023, 0.0034, and 0.0044 W/g, respectively. An upward trend in cumulative hydration heat release with increasing w/b can be seen in Figure 9b. When the w/b was 0.40, 0.45, and 0.50, the cumulative hydration heat release was 203.82, 245.84, and 256.79 W/g, respectively. The results imply that increasing the w/b accelerated heat evolution and promoted early hydration to a certain degree. By contrast, at the lower w/b, the contact between cement particles and CG particles became tighter, which restricted ion diffusion within the system and suppressed both the hydration reaction and the pozzolanic reaction to a certain extent. This ultimately led to a delayed exothermic peak and a reduction in cumulative hydration heat release.

### 4.2. XRD Analysis

**Figure 10:**
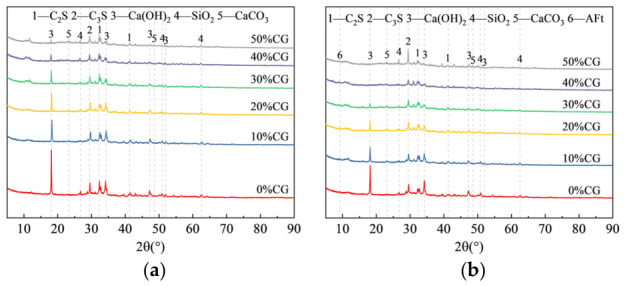
XRD patterns of the CGCC with different CG dosages. (**a**) 3 d. (**b**) 28 d.

Analysis of the CS of the CGCC showed that the system exhibited favorable mechanical performance at w/b 0.40. Figure 10 displays the XRD results of composite cementitious materials with different activated CG dosages at w/b 0.40, and Figure 10a corresponds to the samples cured for 3 d. The main phases in the system included unhydrated C_3_S and C_2_S from the cement clinker minerals, as well as CH produced by cement hydration. As hydration proceeded, the characteristic diffraction peak of ettringite (AFt) appeared at 2θ = 9.19° in Figure 10b. The hydration of C_3_S and C_2_S in cement clinker generated CH, thereby providing an alkaline environment; the corresponding reaction relationships are given in Equations (1) and (2). In addition, the CaCO_3_ peaks observed at 2θ = 23.13° and 48.52° were assigned to the carbonation reaction between CH and CO_2_ in air. The formation of AFt through the reaction between C_3_A and gypsum (CaSO_4_·2H_2_O) in cement can be expressed by Equation (3).

The intensity of the CH diffraction peaks at 2θ = 18.12°, 34.17°, and 47.18° declined progressively as the CG dosage increased, as illustrated in Figure 10a. Under alkaline conditions, CG underwent a pozzolanic reaction and used up CH. In this process, active SiO_2_ reacted with CH to form C-S-H, while active Al_2_O_3_ formed C-A-H through reaction with CH. The corresponding chemical reaction equations are given in Equations (4) and (5). As the pozzolanic reaction continuously consumed CH, the intensity of its diffraction peaks gradually decreased.

(1) $$2C_{3}S+6H_{2}O\to \text{C-S-H}+3CH$$

(2) $$2C_{2}S+4H_{2}O\to \text{C-S-H}+CH$$

(3) $$2C_{3}A+3\left(CaSO_{4}\cdot 2H_{2}O\right)+26H_{2}O\to 3CaO\cdot Al_{2}O_{3}\cdot 3CaSO_{4}\cdot 32H_{2}O(AFt)$$

(4) $$SiO_{2}+CH+H_{2}O\to CaO\cdot SiO_{2}\cdot 2H_{2}O(\text{C-S-H})$$

(5) $$Al_{2}O_{3}+3Ca{\left(OH\right)}_{2}+3H_{2}O\to 3CaO\cdot Al_{2}O_{3}\cdot 6H_{2}O(\text{C-A-H})$$

At 28 d, as shown in Figure 10b, the CH-related diffraction peaks were markedly less intense than those at 3 d. Moreover, when the activated CG content reached 40%, the characteristic CH peak at 2θ = 18.12° disappeared in the XRD pattern of the CGCC. This further confirms that activated CG consumed CH through the pozzolanic reaction in the cement-based system. It can also be observed that the diffraction peaks of AFt in the cementitious systems containing CG were significantly stronger than those in the ordinary Portland cement. This is likely related to the presence of reactive Al_2_O_3_ in the activated CG, which, under the alkaline environment provided by cement hydration, could react with Ca^2+^ in the system and SO_4_^2−^ supplied by gypsum, thereby promoting AFt formation. Activated CG particles also provided extra nucleation sites for hydrate growth, which favored AFt formation and buildup. No diffraction peaks corresponding to the pozzolanic reaction products, namely C-S-H and C-A-H, were observed in the XRD patterns of the CGCCs. This is because both C-S-H and C-A-H possess low crystallinity and therefore cannot be clearly detected by XRD [36].

**Figure 11:**
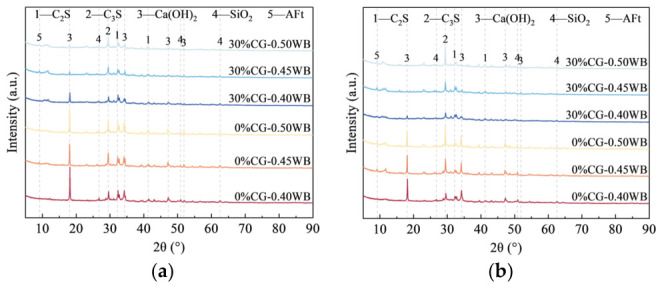
XRD patterns of CGCC at w/b. (**a**) 3 d. (**b**) 28 d.

The phase patterns of the CGCC obtained at different w/b are given in Figure 11. The CH-related diffraction peaks in the 30% CG sample were markedly less intense than those of the ordinary Portland cement paste. This is because the secondary hydration reactions of the active SiO_2_ and active Al_2_O_3_ in the activated CG consumed CH produced by cement hydration and generated secondary hydration products with low crystallinity, thereby reducing the relative CH content in the system. The AFt diffraction intensity in both systems increased overall with increasing w/b, reflecting improved clinker dissolution and accelerated early hydration. In contrast, the diffraction peaks of CH in the 30% CG samples exhibited a weakening trend, suggesting that under higher w/b conditions, the pozzolanic effect of CG was enhanced and accelerated the consumption of CH in the system [37].

### 4.3. FTIR Analysis

**Figure 12:**
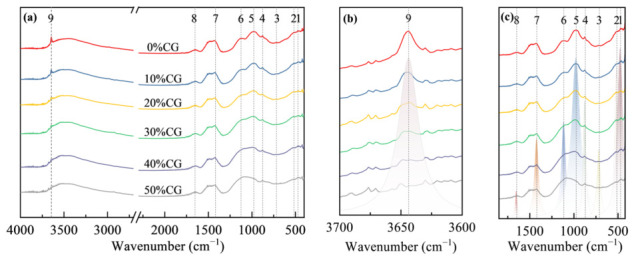
FTIR of CGCC with different CG dosages at 3 d. (**a**) FTIR spectra of 500~4000 cm^−1^; (**b**) Spectral peak fitting of 3600~3700 cm^−1^; (**c**) Spectral peak fitting of 400~1800 cm^−1^.

The FTIR spectra of the composite cementitious materials with varying CG contents at 3 d are shown in Figure 12, while the peak assignments and corresponding vibrational and phase information are given in Table 4. For cement paste, spectral peak fitting was conducted within 3600–3700 cm^−1^ and 400–1800 cm^−1^, and the deconvoluted results are displayed in Figure 12b,c, respectively. The findings suggest that, with increasing activated CG content, the characteristic O-H peak gradually weakened, and it nearly disappeared when the CG content reached 50%. This finding suggests that the active SiO_2_ and active Al_2_O_3_ in the activated CG continuously consumed CH and promoted the formation of secondary hydration products, and that the higher the CG content, the more pronounced the consumption of CH.

**Figure 13:**
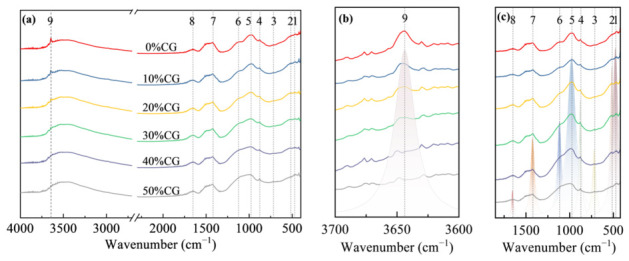
FTIR of the CGCC with different CG dosages at 28 d. (**a**) FTIR spectra of 500~4000 cm^−1^; (**b**) Spectral peak fitting of 3600~3700 cm^−1^; (**c**) Spectral peak fitting of 400~1800 cm^−1^.

The FTIR spectra of the CGCC at 28 d are shown in Figure 13. By comparing the characteristic O-H peaks at 3 d and 28 d, a pronounced weakening of the O-H peak was observed as hydration progressed, indicating the continuous consumption of CH in the system. A major reason is that ongoing cement hydration continuously releases CH, maintaining pore solution alkalinity and facilitating the pozzolanic reaction of CG. Consequently, the reactive SiO_2_ and Al_2_O_3_ remained involved in secondary reactions, which progressively lowered the relative amount of CH in the system.

A rise-fall trend with increasing CG content was observed for the 977 cm^−1^ peak corresponding to [SiO_4_] tetrahedral vibration in C-(A)-S-H. The result suggests an initial rise in C-(A)-S-H formation as the CG level rose. However, as a result of the elevated specific surface area of CG, excessive water was absorbed by the CG during mixing, resulting in insufficient cement hydration and thus a reduced generation of CH. This likely restricted the secondary reaction of CG with CH, thereby decreasing C-(A)-S-H generation. Furthermore, when an activated CG content was excessively high, the compactness of the matrix decreased. Under such conditions, CH was more likely to react with CO_2_ in air, resulting in an insufficient amount of CH available for the pozzolanic reaction, thereby hindering the formation of C-(A)-S-H. This trend is consistent with the deterioration in macroscopic strength.

**Figure 14:**
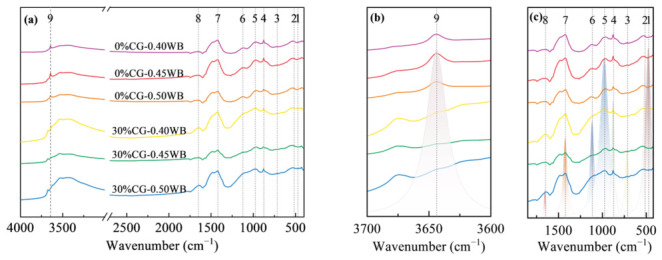
FTIR of the CGCC at 3 d under different w/b. (**a**) FTIR spectra of 500~4000 cm^−1^; (**b**) Spectral peak fitting of 3600~3700 cm^−1^; (**c**) Spectral peak fitting of 400~1800 cm^−1^.

**Figure 15:**
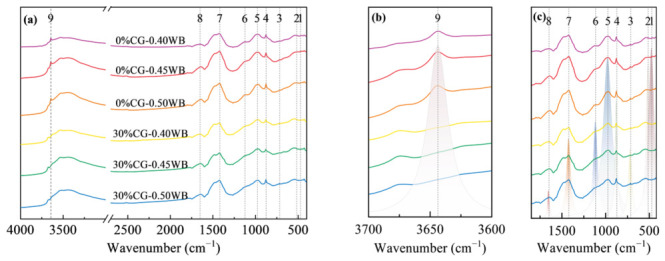
FTIR of CGCC at 28 d under different w/b. (**a**) FTIR spectra of 500~4000 cm^−1^; (**b**) Spectral peak fitting of 3600~3700 cm^−1^; (**c**) Spectral peak fitting of 400~1800 cm^−1^.

Figure 14 and Figure 15 show the FTIR spectra of the CGCC at different w/b. The CGCC showed enhanced peaks at 875 and 1420 cm^−1^ as the w/b rose from 0.40 to 0.50; these bands are associated with CO_3_^2−^ out-of-plane bending and symmetric C-O stretching in CaCO_3_, respectively. A possible explanation is that increasing the w/b made the matrix less compact and weakened particle bonding, allowing CO_2_ to penetrate more easily and react with CH, which favored CaCO_3_ generation.

### 4.4. TG/DTG Analysis

**Figure 16:**
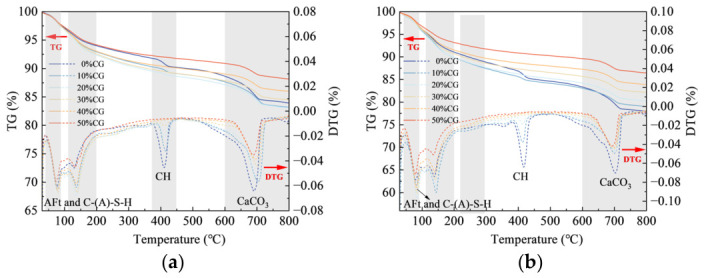
TG/DTG curves of CGCC with different CG. (**a**) 3 d. (**b**) 28 d.

The TG/DTG curves of CGCC with different activated CG contents are displayed in Figure 16. The TG traces record weight loss in different temperature regions, while the DTG signals correspond to the decomposition of various hydration phases [45]. Four characteristic DTG peaks can be identified in the CGCC cured for 3 d and 28 d. Among them, the peak between 50 and 200 °C mainly corresponded to dehydration of AFt and C-(A)-S-H [19,46]. The peak observed between 375 and 450 °C mainly corresponded to CH decomposition [47]. The mass reduction in the 600–800 °C interval mainly resulted from CaCO_3_ decarbonation [48].

According to Figure 16a, at the age of 3 d, the DTG peak corresponding to CH gradually weakened with increasing activated CG content, indicating that the CH content in the cementitious system decreased as the CG dosage increased. This was mainly because the active SiO_2_ and Al_2_O_3_ in the CG underwent pozzolanic reactions in the alkaline environment and consumed CH, generating secondary hydration products with low crystallinity and thereby weakening the decomposition peak of CH. Table 5 and Table 6 present the calculated mass losses of chemically bound water and CH in the samples at ages of 3 and 28 d, respectively. At 3 d, when the activated CG content was 10%, 20%, 30%, 40%, and 50%, the mass losses of CH in the CGCC were 1.08%, 0.57%, 0.46%, 0.28%, and 0.09%, respectively. These values were 0.37%, 0.88%, 0.99%, 1.17%, and 1.36% lower than those of the 0% CG sample, respectively. Meanwhile, the strongest DTG signals were observed for AFt and C-(A)-S-H, suggesting that cement hydrates and pozzolanic products derived from CG were the dominant phases in the system and played a major role in building the matrix structure. With increasing CG content, the DTG peak intensity corresponding to C-(A)-S-H and AFt first increased and then decreased. With an appropriate amount of CG added, both AFt formation from hydration and C-(A)-S-H generation from pozzolanic activity were facilitated. However, when the activated CG content exceeded 30%, the contents of C-(A)-S-H and AFt began to decrease markedly. With less cement clinker available, the system contained fewer reactive phases and less CH, restricting CG pozzolanic reaction and thus lowering the amounts of C-(A)-S-H and AFt formed.

According to Figure 16b, the CH-related DTG peak at 28 d declined continuously as the CG level increased. The mass losses of CH in the cementitious materials containing 0%, 10%, 20%, 30%, 40%, and 50% CG were 1.96%, 1.02%, 0.55%, 0.29%, 0.19%, and 0.03%, respectively. The mass loss of CH in the 0% CG sample at 28 d was 0.51% higher than that at 3 d, indicating that as hydration proceeded, hydration products continuously accumulated, leading to an increase in CH content and thus a higher mass loss. In comparison, the CH level in the samples containing CG was much lower at 28 d than at 3 d, suggesting strong pozzolanic activity of the activated CG. As the secondary reaction of CG proceeded, CH was continuously depleted, more gel-like products were generated, and the microstructure became increasingly refined.

**Figure 17:**
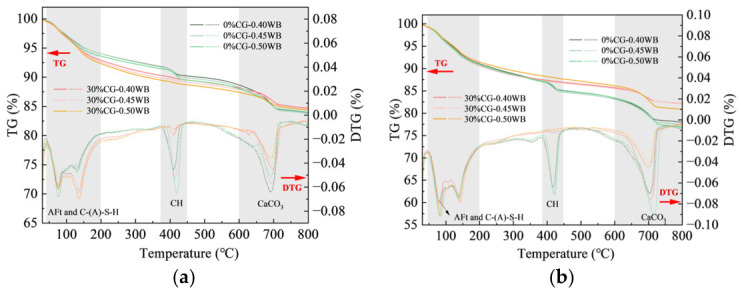
TG/DTG curves of CGCC at different w/b. (**a**) 3 d. (**b**) 28 d.

Figure 17 shows the TG/DTG results of CGCC prepared at different w/b. In Figure 17a, the DTG peak assigned to CH decomposition between 375 and 450 °C is clearly less intense in the 30% CG specimen than in the 0% CG specimen, indicating reduced CH content after the introduction of activated CG. A likely reason is that reactive SiO_2_ and Al_2_O_3_ in activated CG consumed CH produced during cement hydration through pozzolanic reaction, leading to the formation of more secondary hydrates such as C-(A)-S-H gel. In parallel, increasing the w/b caused the CH peak in the 0% CG samples to become stronger, while the corresponding peak in the 30% CG samples generally weakened.

Table 7 and Table 8 give the mass-loss results of chemically bound water and CH for the 0% CG and 30% CG mixtures under different w/bs at 3 d and 28 d, respectively. For both mixtures, chemically bound water increased noticeably with curing age. The result suggests continuous progress of cement hydration and CG secondary reaction with curing time, accompanied by ongoing formation of AFt and C-(A)-S-H. The 30% CG specimens consistently showed greater chemically bound water contents than the corresponding 0% CG group at both curing ages. Taking the 3 d results as an example, the chemically bound water contents of the 0% CG–0.40 WB, 0% CG–0.45 WB, and 0% CG–0.50 WB groups were 5.68%, 5.70%, and 5.93%, respectively, whereas the corresponding values for the 30% CG samples increased to 6.63%, 6.96%, and 7.16%, respectively. These results indicate that activated CG not only exerted a nucleation effect but also participated in secondary hydration through the pozzolanic reaction, thereby promoting the formation of gel phases.

Furthermore, for the 0% CG system, with increasing w/b, the chemically bound water content increased from 5.68% to 5.93% at 3 d and from 8.37% to 8.58% at 28 d, showing an overall increasing trend. Meanwhile, the CH content increased from 1.64% to 2.02% at 3 d and from 2.11% to 2.27% at 28 d. This implies that, for the pure cement system, a higher w/b improves particle dispersion and ion transport conditions, thereby promoting the hydration of clinker minerals and increasing the generation of hydration products. By contrast, for the cementitious system containing 30% CG, the CH content showed a decreasing trend with increasing w/b. The result indicates that increasing the w/b promoted both cement hydration and the pozzolanic reaction of CG, leading to more effective CH consumption. Even though CH was consumed more extensively as the w/b increased, the extra free water left behind more pores after hardening, which weakened the mechanical properties of the material.

### 4.5. Pore Structure Analysis

**Figure 18:**
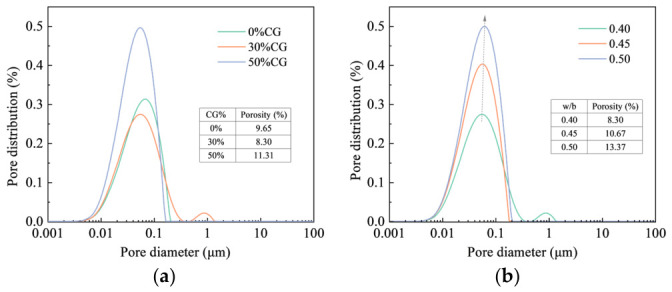
Evolution of the pore structure of CGCC. (**a**) Effect of CG content. (**b**) Effect of w/b.

Figure 18 presents the evolution of the pore structure of CGCC. As shown in the pore-size distribution curves, the main peak is primarily concentrated within the range of 0.01~0.1 μm.

According to Figure 18a, the porosity of CGCC first decreases and then increases with the rise in CG content. When the CG content reaches 30%, the porosity attains its minimum value, indicating that the pozzolanic reaction of CG generates C-(A)-S-H gels that densify the CGCC matrix. This trend is consistent with the observed variation in compressive strength. Figure 18b shows that the porosity of CGCC increases with increasing w/b. Meanwhile, the main peak gradually shifts toward the right as the w/b increases (as shown by the dashed line), suggesting that a higher w/b introduces excess water into the system, which subsequently leads to larger pore sizes. Combined with the CS results, it can be inferred that increasing the w/b reduces the compactness of the CGCC matrix, ultimately resulting in lower CS.

### 4.6. SEM Analysis

**Figure 19:**
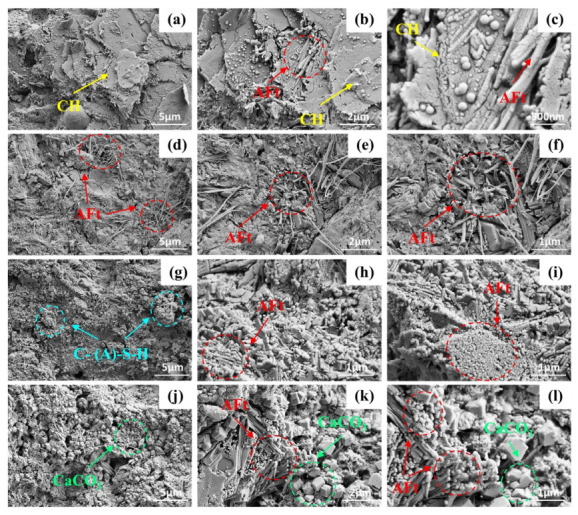
SEM images with different CG content: (**a**–**c**) 0% CG; (**d**–**f**) 10% CG; (**g**–**i**) 30% CG; (**j**–**l**) 50% CG.

Figure 19 shows the SEM micrographs of the composite cementitious materials with varying activated CG dosages at 28 d. Figure 19a–c presents the microstructure of the 0% CG cement paste, in which typical plate-like CH crystals and fibrous AFt crystals can be clearly observed [42]. Figure 19d–f shows the microstructure of the 10% CG sample, where a relatively large amount of interwoven fibrous AFt can be observed, filling the pores to a certain extent and resulting in a denser matrix morphology. For the 30% CG sample in Figure 19g–i, AFt appears in dense clusters, accompanied by abundant flocculent C-(A)-S-H gel. Under alkaline conditions, reactive SiO_2_ and Al_2_O_3_ in activated CG reacted with CH to produce this gel as a secondary hydration product. These products could fill pores and enhance matrix continuity, thereby contributing positively to strength development.

When the activated CG content increased to 50%, Figure 19j–l shows that the matrix structure became looser, and cubic CaCO_3_ crystals could be observed. A possible explanation is that the reduced cement content at high CG dosage, which led to insufficient generation of hydration products and thus restricted the secondary reaction of activated CG and the formation of gel products, ultimately weakening matrix densification. Meanwhile, the looser structure made CH more likely to come into contact with CO_2_ in the air and undergo carbonation, resulting in the formation of CaCO_3_ crystals.

**Figure 20:**
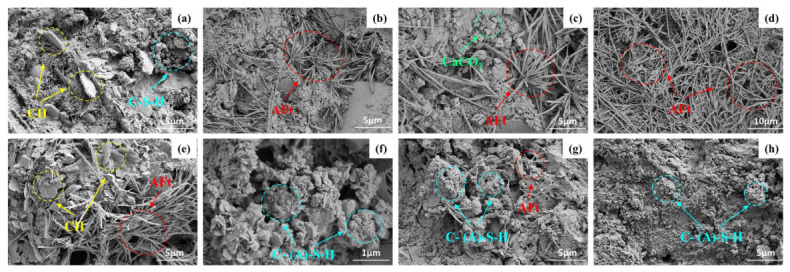
SEM micrographs of the CGCC at different hydration ages: (**a**–**d**) 0% CG at 3, 7, 14, and 28 d; (**e**–**h**) 30% CG at 3, 7, 14, and 28 d.

SEM micrographs of CGCC at different hydration ages are given in Figure 20. For the 0% CG specimens in Figure 20a–d, AFt became increasingly abundant as hydration continued. By 28 d, a large amount of AFt had interwoven into a network-like framework that, along with the gel phase, formed the main skeleton of the matrix. Figure 20e–h shows the microstructures of the 30% CG samples at different ages. At 3 d, plate-like CH crystals and fibrous AFt could be observed. As curing progressed, CG persistently participated in pozzolanic reactions under alkaline conditions, leading to CH consumption and C-(A)-S-H formation. At 28 d, a large amount of flocculent C-(A)-S-H was observed filling the pore structure and the interstitial spaces between crystals, which promoted matrix continuity and densification, thereby making a significant contribution to strength enhancement.

**Figure 21:**
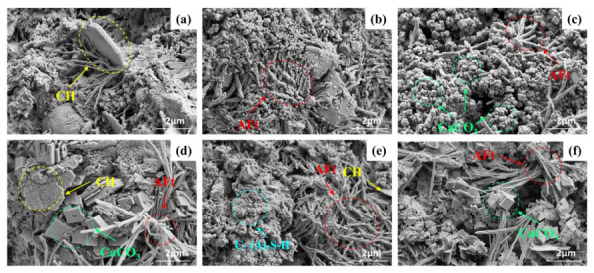
SEM micrographs of CGCC at different w/bs: (**a**–**c**) 0% CG at w/b = 0.40, 0.45, and 0.50; (**d**–**f**) 30% CG at w/b = 0.40, 0.45, and 0.50.

Figure 21 presents SEM images of CGCC with 0% and 30% CG under different w/bs. The microstructures of the 0% CG samples at w/bs of 0.40, 0.45, and 0.50 are shown in Figure 21a–c, respectively. At a w/b of 0.40, CH and AFt were found to be interwoven within the cementitious material, and the matrix exhibited a relatively dense overall structure. When the w/b increased to 0.50, the paste exhibited a relatively loose microstructure. Under such conditions, external CO_2_ could more readily penetrate through the pore channels and react with CH, thereby leading to the formation of CaCO_3_ crystals. For the 30% CG specimens, Figure 21d–f compares the SEM images at w/bs of 0.40, 0.45, and 0.50. At the highest w/b, the microstructure became noticeably porous, and accumulated CaCO_3_ crystals were also visible. At higher w/bs, excess free water in the system was more likely to produce additional pores after hardening. Meanwhile, carbonation in the system became more pronounced, which was unfavorable for the densification and strength development of the composite cementitious system.

## 5. Conclusions

The effects of CG content and w/b on the mechanical performance, hydration behavior, and microstructural evolution of CGCC were investigated in this work, and the main findings are summarized below.

(1) The CS of the CGCC first increased and then decreased with increasing CG content. At a CG content of 30%, the material showed the best mechanical response, achieving a 28 d CS of 59.1 MPa, which was 18.9% above that of the CG-free sample.
(2) As the CG content increased, hydration and pozzolanic reactions became more pronounced, leading to lower CH levels and greater formation of AFt and C-(A)-S-H, which benefited mechanical performance.
(3) An increase in w/b resulted in lower CS of the CGCC, whereas decreasing the ratio from 0.50 to 0.40 improved strength by 44.4%.
(4) Adjusting the w/b between 0.40 and 0.50 did not affect the types of hydration products present, but it did slightly enhance the early hydration process in the CGCC. Nevertheless, excess free water reduced matrix compactness after hardening, which was unfavorable for the development of CS.

## 6. Research Limitations and Recommendations for Future Work

In this study, CG was used as a partial replacement for cement to investigate its effects on the mechanical properties and microstructural characteristics of the cementitious system, thereby elucidating the strength-development mechanism of CGCC. However, the scope of this work remains limited to the behavior of the cementitious matrix itself and has not yet been extended to concrete-level performance. Existing studies have demonstrated that CG can undergo pozzolanic reactions with cement hydration products. The reactive aluminosilicate phases in CG react with calcium hydroxide to form C-A-S-H gels, which contribute to pore refinement and microstructural densification. The resulting reduction in pore connectivity and the formation of a denser pore network can significantly inhibit the transport of aggressive ions such as chloride and sulfate, thereby slowing deterioration processes and enhancing durability in corrosive environments. Based on these insights, future research should further investigate the durability performance of activated CG-based materials under various aggressive conditions. Such studies will provide theoretical support for the design and application of CG-based materials in complex service environments.

## Figures and Tables

**Table 1:** Oxide composition (wt.%).

Composition	CaO	SiO_2_	Al_2_O_3_	MgO	Fe_2_O_3_	SO_3_	TiO_2_	K_2_O	Na_2_O	Others
Cement	46.72	24.63	12.18	4.83	4.63	3.29	1.11	1.26	1.14	0.21
CG	3.36	43.63	48.04	0.46	0.42	1.24	0.28	0.42	/	2.15

**Table 2:** Particle size parameters of CG and cement (μm).

Particle Size	D10	D15	D25	D40	D50	D60	D75	D85	D90
Cement	3.24	4.75	7.79	12.64	16.19	20.18	28.01	36.08	42.16
CG	1.53	2.22	4.08	8.28	12.29	16.98	26.27	34.51	41.46

**Table 3:** Mix proportion of CGCC.

No.	CG Content (wt.%)	Cement Content (wt.%)	w/b	Age
0% CG–0.40 WB	0	100	0.40	3, 7, 14, 28 d
10% CG–0.40 WB	10	90		
20% CG–0.40 WB	20	80		
30% CG–0.40 WB	30	70		
40% CG–0.40 WB	40	60		
50% CG–0.40 WB	50	50		
0% CG–0.45 WB	0	100	0.45	3, 7, 14, 28 d
10% CG–0.45 WB	10	90		
20% CG–0.45 WB	20	80		
30% CG–0.45 WB	30	70		
40% CG–0.45 WB	40	60		
50% CG–0.45 WB	50	50		
0% CG–0.50 WB	0	100	0.50	3, 7, 14, 28 d
10% CG–0.50 WB	10	90		
20% CG–0.50 WB	20	80		
30% CG–0.50 WB	30	70		
40% CG–0.50 WB	40	60		
50% CG–0.50 WB	50	50		

**Table 4:** Characteristics of chemical bonds in CGCC.

Phase	Functional Group	Wavenumber (cm^−1^)	No.	Reference
SiO_2_	Symmetric stretching vibration of Si-O-Si	470, 715	1, 3	[38]
C_2_S	Bending vibration of Si-O-Si	516	2	[39]
CaCO_3_	Out-of-plane bending vibration of CO_3_^2−^	875	4	[40]
	Symmetric stretching vibration of C-O	1420	7	[36]
C-(A)-S-H	[SiO_4_] tetrahedral vibration	977	5	[41]
	H-O-H bending vibration	1653	8	[42]
AFt	S-O stretching vibration	1122	6	[43]
CH	O-H stretching vibration	3644	9	[44]

**Table 5:** Mass loss of each phase in CGCC at 3 d.

CG Content	Mass Loss of Chemically Bound Water (wt.%)			Mass Loss of CH (wt.%)		
	W_50 °C_	W_200 °C_	ΔW(H_2_O)	W_375 °C_	W_450 °C_	ΔW(CH)
0%	99.74	93.99	5.75	91.76	90.31	1.45
10%	99.64	92.93	6.71	90.16	89.08	1.08
20%	99.51	92.36	7.15	89.38	88.73	0.57
30%	99.56	92.55	7.01	89.66	89.20	0.46
40%	99.54	93.04	6.50	90.57	90.29	0.28
50%	99.59	94.16	5.43	91.94	91.85	0.09

**Table 6:** Mass loss of each phase in the CGCC at 28 d.

CG Content	Mass Loss of Chemically Bound Water (wt.%)			Mass Loss of CH (wt.%)		
	W_50 °C_	W_200 °C_	ΔW(H_2_O)	W_375 °C_	W_450 °C_	ΔW(CH)
0%	99.40	91.07	8.33	87.17	85.21	1.96
10%	99.15	89.91	9.36	85.79	84.77	1.02
20%	99.15	89.79	9.36	86.30	85.75	0.55
30%	99.36	90.59	8.77	87.43	87.14	0.29
40%	99.29	91.53	7.76	88.57	88.38	0.19
50%	99.46	93.17	6.29	90.74	90.61	0.03

**Table 7:** Mass loss of each phase in CGCC at 3 d for w/bs.

w/b	Mass Loss of Chemically Bound Water (wt.%)			Mass Loss of CH (wt.%)		
	W_50 °C_	W_200 °C_	ΔW(H_2_O)	W_375 °C_	W_450 °C_	ΔW(CH)
0% CG–0.40 WB	99.73	94.05	5.68	91.81	90.17	1.64
0% CG–0.45 WB	99.60	93.90	5.70	91.72	89.86	1.86
0% CG–0.50 WB	99.50	93.57	5.93	91.45	89.43	2.02
30% CG–0.40 WB	99.50	92.87	6.63	90.28	89.43	0.85
30% CG–0.45 WB	99.52	92.56	6.96	89.80	89.18	0.71
30% CG–0.50 WB	99.45	92.29	7.16	89.46	88.75	0.62

**Table 8:** Mass loss of each phase in CGCC at 28 d for w/bs.

w/b	Mass Loss of Chemically Bound Water (wt.%)			Mass Loss of CH (wt.%)		
	W_50 °C_	W_200 °C_	ΔW(H_2_O)	W_375 °C_	W_450 °C_	ΔW(CH)
0% CG–0.40 WB	99.42	91.05	8.37	87.18	85.07	2.11
0% CG–0.45 WB	99.19	90.62	8.57	86.97	84.73	2.24
0% CG–0.50 WB	99.34	90.76	8.58	87.22	84.95	2.27
30% CG–0.40 WB	99.31	90.68	8.63	87.50	86.94	0.56
30% CG–0.45 WB	99.24	90.53	8.71	87.31	86.71	0.60
30% CG–0.50 WB	99.34	91.47	7.87	88.26	87.72	0.54

## Data Availability

The data presented in this study are available on request from the corresponding authors.
